# Ex Vivo and In Vivo Assessment of the Penetration of Topically Applied Anthocyanins Utilizing ATR-FTIR/PLS Regression Models and HPLC-PDA-MS

**DOI:** 10.3390/antiox9060486

**Published:** 2020-06-03

**Authors:** Alexandra Westfall, Gregory T. Sigurdson, Luis E. Rodriguez-Saona, M. Mónica Giusti

**Affiliations:** Department of Food Science and Technology, The Ohio State University, 2015 Fyffe Ct., Columbus, OH 43210-1007, USA; Westfall.125@buckeyemail.osu.edu (A.W.); Sigurdson.5@buckeyemail.osu.edu (G.T.S.); Rodriguez-Saona.1@osu.edu (L.E.R.-S.)

**Keywords:** anthocyanins, dermal delivery system, color cosmetics, skin barrier, spectroscopy, delivery/vectorization/penetration

## Abstract

Anthocyanins are natural colorants with antioxidant properties, shown to inhibit photoaging reactions and reduce symptoms of some skin diseases. However, little is known about their penetration through the stratum corneum, a prerequisite for bioactivity. The aim was to investigate anthocyanin penetration from lipophilic cosmetic formulations through the skin using a porcine ear model and human volunteers. ATR-FTIR/PLS regression and HPLC-PDA-MS were used to analyze anthocyanin permeation through the stratum corneum. Penetration of all anthocyanins was evident and correlated with molecular weight and hydrophilicity. Lower-molecular-weight (MW) anthocyanins from elderberry (449–581 Da) were more permeable within the skin in both ex vivo and in vivo models (K_p_ = 2.3–2.4 × 10^−4^ cm h^−1^) than the larger anthocyanins (933-1019 Da) from red radish (K_p_ = 2.0–2.1 × 10^−4^ cm h^−1^). Elderberry and red radish anthocyanins were found at all levels of the stratum corneum and at depths for activity as bioactive ingredients for skin health.

## 1. Introduction

In recent years, the health-promoting properties of flavonoids have made them attractive functional ingredients for both the pharmaceutical and cosmetic industries [1]. Anthocyanins are a pigmented subclass of flavonoids of ≥700 structures identified. They are increasing in popularity in the food industry but have been limitedly investigated for bioactive use in topical formulations [2]. In the gastrointestinal tract, they have been shown to potentially act as chemopreventive by preventing oxidative damage when in contact with tissues [3]. Anthocyanins have been investigated for their antioxidant properties when incorporated into emulsions for topical application in vitro [4,5]. They have also shown potential to prevent oxidative damage to the skin by UV-induced erythema, skin cancer, and photoaging in vitro and in vivo [6,7,8,9]. When incorporated into gold nanoparticles, they also demonstrated some activity to improve psoriatic lesions in vitro and alleviate atopic dermatitis in vivo [10,11]. In order for the biological activity of these ingredients to be effective, they must be released from the topically applied formulations, reach the skin, and overcome the barrier function of the stratum corneum (SC) to penetrate into the epidermis and dermis [12].

The release of active ingredients and skin penetration kinetics are dependent on the molecular properties of the compound, such as molecular weight and lipophilicity, as well as the properties of the vehicle [13]. The skin permeation activity of emulsions containing polyphenolic compounds, such as protocatechuic acid, chlorogenic acid, catechin, resversatrol, rutin, quercetin, and epigallocatechin gallate, has previously been investigated [14,15,16,17,18,19]. Although chemically similar, the permeation behavior of each flavonoid varies due to differences in their molecular properties and also vehicle formulation. Few studies have investigated the protective effects of anthocyanins when incorporated into matrices for topical delivery. However, two recent studies showed antioxidant and antityrosinase activities in vitro when anthocyanins were concentrated onto protein-rich matrices [20] and also high retention of antioxidant activity when incorporated into ultradeformable liposomes [21]. The results of these studies were promising; however, the technology utilized does not easily translate into practical or commonly used delivery systems for bioactive ingredients, such as incorporation into pre-existing cosmetic formulas. We recently demonstrated that anthocyanins have high stability when incorporated into a lipstick formulation and that they also exhibited potential bioactive properties when evaluated in vitro [22,23].

Therefore, the objective of this study was to investigate the release of anthocyanins from lipophilic cosmetic formulations, as a novel system of delivery for anthocyanins, and their skin permeation behavior using both ex vivo porcine skin and in vivo human skin methods. Elderberry, a source of non-acylated cyanidin, and red radish, a source of acylated pelargonidin derivatives, were used in this investigation. These plant materials were chosen as sources as anthocyanins to help elucidate the role that different structural attributes on anthocyanins, including glycosylation and acylation patterns, may have on skin permeation behavior.

## 2. Materials and Methods 

### 2.1. Materials 

Elderberry and red radish dried extracts were provided by DD Williamson & Co., Inc. (Louisville, KY, USA). The base of the lipstick formulations was purchased from MakingCosmetics, Inc. (Snoqualmie, WA, USA); ingredients for the base were triglyceride, coconut oil, octyldodecanol, ozokerite wax, polyisobutene, castor oil, isopropyl palmitate, microcrystalline wax, lanolin oil, microcrystalline wax, synthetic wax, glycerin, dl-alpha tocopherol, and butylated hydroxytoluene (BHT). Adhesive tape D-Squame disks (CuDerm Corportation, Dallas, TX, USA) with a diameter of 2.2 cm were used for tape stripping. Bovine serum albumin (BSA) standard was purchased from BioRad (Bio-Rad DC, Hercules, CA, USA).

### 2.2. Porcine Skin Tissue

Pig ears were obtained from a local abattoir (Massillon, OH, USA) and stored at −24 °C for no more than 6 months prior to use. The pig ears were thawed and cleaned with cold water and then gently dried with a soft tissue. Full-thickness skin was removed from the cartilage with a scalpel, and any hair was removed. Samples were then cut into 2.5 × 7.5 cm pieces and pinned to a Styrofoam board with needles in each corner. 

### 2.3. Subject Recruitment

Approval for the human study protocol was received from the Institutional Review Board for Human Research of the Ohio State University (IRB# 2014H0161). Six healthy female volunteers (Fitzpatrick skin types III-IV), aged 22–27 years old, with no known history of dermatological diseases or drug allergy, were included in the study. Participants were required to not apply any topical drugs or cosmetics to the test sites at least 12 h before the experiment.

### 2.4. Formulations

Lipstick formulations were prepared using the commercially available base described above, with the anthocyanin extracts comprising 8% (w/w) of the final formulation according to our previous studies [22]. This percentage was chosen in accordance with current recommendations for lipstick colorants used by the cosmetic industry [24].

### 2.5. Tape Stripping Procedures

Lipstick, with and without anthocyanins, was applied to porcine ear skin samples at 2 mg/cm^2^. The porcine ear samples were then incubated at 32 °C for 1 h (Isotemp Incubator. Fisher Scientific. Hampton, NH, USA). After incubation, the lipstick remaining on the skin surface was removed by blotting with a tissue. Adhesive tape was then applied to the skin sample with a rolling motion of the thumb and removed in one continuous motion. Ten sequential tape strips were applied and removed in the same manner. Samples were also collected of porcine ear skin without application of lipstick in order to eliminate error sources caused by variations in stratum corneum composition between the samples. The tape strips were then carefully cut in half and each half was used either for analysis by both high-performance liquid chromatography (HPLC) or by ATR-FTIR.

The in vivo clinical study was performed in 2 trials in which the lipstick formulations were applied to subjects in different patterns, allowing for evaluation of anthocyanin penetration as well as their transversal diffusion. The four test sites were 1 in^2^ in size with 1 in distance between test sites on the volar forearms of the subjects. In Trial 1, the 2 lipstick formulations were applied in alternating pattern to each of the test sites; the order of application was randomized. In Trial 2, each of the lipstick formulations was applied to 1 test site that were alternated between the other 2 test sites which remained untreated. Lipstick formulations were applied at 2 mg/cm^2^. All experimental sites were left unoccluded for 1 h after the application. Residual lipstick was removed and tape stripping was conducted as described for the porcine skin model. Control samples were collected from the forearm approximately 2 in away from nearest test sites. 

Lipstick formulations containing anthocyanin extracts at concentrations of 0–8% and also bovine serum albumin (BSA) at concentrations of 0–500 μg were also analyzed. All experiments were performed in triplicate (*n* = 3).

### 2.6. Analysis of Removed Tape Strips

#### 2.6.1. Attenuated Total Reflectance Fourier Transform Infrared Spectroscopy

Attenuated total reflectance (ATR)-FTIR spectra were collected of the tape strips with the adhesive side facing the ZnSe ATR Crystal of the Excalibur FT-IR 3100 spectrometer (Varian Technologies, Palo Alto, CA, USA). Spectra were collected over the range of 4000–700 cm^−1^ at 4 cm^−1^ resolution. Interferograms of 64 scans were coadded followed by Beer–Norton apodization. The absorbance spectra were ratioed against the blank ATR crystal spectrum collected as background. 

#### 2.6.2. HPLC-PDA-MS Analysis

Contents of the recovered anthocyanins from the tape strips were determined by HPLC. Individual cut tape strips were submerged in 1.0 mL extraction solvent (50:50 MeOH: acidified distilled deionized H_2_O (0.01% HCl) in 2 mL centrifuge tubes. The centrifuge tubes were then shaken at room temperature for 1 hr at 40 strokes/minute, followed by bath sonication for 20 min. Nitrogen flushing was used to evaporate off the methanolic fraction of the solvent, and samples were brought up to a 1.0 mL volume in acidified distilled deionized water (0.01% HCl).

A reverse-phase high-performance liquid chromatography (HPLC) system (Shimadzu Corporation, Columbia, MD, USA) consisting of a LC-20AD prominence liquid chromatograph, a SPD-M20A prominence diode array (PDA) detector coupled with a LCMS-2010 Mass Spectrometer (Shimadzu Corporation, Columbia, MD, USA) was utilized. LCMS solution Ver 3.30 software was used. A reverse-phase 3.5 µm Symmetry C18 column (4.6 × 150 mm, Waters Corp., MA, USA) fitted with a 4.6 × 150 mm Symmetry 5 microguard column (Waters Corp., MA, USA) was used. Solvents and samples were filtered through 0.45 µm polypropylene membrane filters. Separation was achieved by using the following binary gradient mobile phase with solvent A as 4.5% (v/v) formic acid in water and B as 100% acetonitrile: 0–5 min at 7% B; 5–20 min for 7–20% B, 20–30 min for 20–40% B; 30–40 min for 40–7% B; and 45–45 min at 7% B. A 0.8 mL/min flow rate was used, and injection volumes were 30 µL. Spectral information was collected from 260 to 700 nm, and elution was monitored at 280 and 520 nm.

The anthocyanins in all samples were quantified based on a standard curve of the absorbance under the curve (520 nm) developed from solutions of known amounts of cyanidin-3-glucoside standard from 0–100 μg/mL (*R*^2^ = 0.999). The limit of detection (LOD) and limit of quantitation (LOQ) were based on 3× and 10× signal to noise ratio, respectively. Untreated skin samples were analyzed under the same conditions and served as a baseline for comparison.

For MS analysis, 0.2 mL/min flow was diverted to the MS and ionized under positive ion conditions using an electrospray probe (ESI). Data was initially monitored using a total ion scan from *m/z* 200–1200 and then with selective ion monitoring at *m/z* 271 (pelargonidin), *m/z* 287 (cyanidin), *m/z* 301 (peonidin), *m/z* 303 (delphinidin), *m/z* 317 (petunidin), and *m/z* 331 (malvidin).

### 2.7. Chemometrics

Multivariate statistical software (Pirouette 4.2, Infometrix Inc., Woodinville, WA, USA) was used to collect information from the IR spectra and to conduct data reduction analysis for statistical analysis purposes. In order to mitigate the influence of variations in signal intensity and to resolve overlapping bands, spectra were normalized and second derivative transformed using a five-point polynomial-fit Savitsky–Golay function, respectively.

For the stratum corneum samples collected, the Amide II absorbance was used as an internal standard to account for variations in sample contact with the ATR crystal [25]. Partial least squares (PLS), a multivariate regression, was used to develop a calibration model of anthocyanin-lipstick formulation at varying concentrations based on FT-IR spectra to predict the relative amounts of anthocyanins present in the tape strips acquired from the porcine and human skin. Cross-validation using the leave-one-out approach was used to obtain the calibration model.

### 2.8. Protein Quantification

The amount of corneocytes removed from the individual tape strips after the incubation period was determined by linear regression of the characteristic N-H bending vibrations of Amide I and Amide II absorbance peaks (~1640 cm^−1^ and ~1540 cm^−1^) of the BSA concentration curve (*R*^2^ = 0.98). The absorptions were normalized for a tape area of 1 cm^2^ and expressed as μg/cm^−2^. The mean protein density was assumed to be 1 g/cm^−3^ and was used to determine the thickness of the stratum corneum [26]. Due to the inherent variability between porcine ear skin thicknesses, six replicate experiments were performed to ensure the obtained values were in agreement with the literature. The mean stratum corneum thickness of the porcine ear skin was determined to be 8.3 ± 2.4 μm. The determined value was in agreement with the assumed thickness of porcine ear stratum corneum of 8.2–8.5 ± 3.0 μm [27,28]. The mean stratum corneum thickness from the clinical trials was determined to be 13.69 ± 2.61 μm [27,29]. 

In general, the porcine skin without lipstick treatment showed the highest amount of removed corneocytes after tape stripping. This may be due to decreased hydration of the skin relative to the samples with lipstick applied to them [25]. Another possible reason for the decreased amount of corneocytes removed for the skin samples with lipstick applied could be due to the interaction of the adhesive on the tape strips with the lipstick ingredients, which would decrease the amount of corneocytes removed [30].

### 2.9. Determination of Anthocyanin Penetration Profiles

Permeation kinetics of the anthocyanins in the lipstick and also through the stratum corneum were determined after the thickness of the stratum corneum removed by each tape strip and the amount of anthocyanin on each tape strip were normalized to the tape strips’ areas [13]. Fick’s second law of diffusion was used to predict concentrations of anthocyanin (c_x_) as a function of position (x) within the stratum corneum (See Appendix A) [31]. Fick’s first law was used to determine initial release rates of the anthocyanins from the formulations (See Appendix A). Likewise, Higuchi’s kinetic model was used to understand the influence of the anthocyanin non-initial release kinetics from the lipstick formulas on the permeation kinetics within the SC (See Appendix A).

## 3. Results and Discussion

### 3.1. Characterization of the Anthocyanins in Elderberry and Red Radish Lipstick Formulations

The elderberry extract consisted of two major anthocyanins, cyanidin-3-sambubioside (Cy-3-samb) and cyanidin-3-glucoside (Cy-3-glu), with molecular weights of 581.5 and 449.2 Da, respectively (Table 1) [32]. Cy-3-samb accounted for ~60% of the total anthocyanins present in the extract, and Cy-3-glu accounted for ~37%, consistent with the literature [32,33]. Anthocyanins from red radish were predominantly derivatives of pelargonidin-sophoroside-5-glucoside (Pg-soph-5-glu) acylated with cinnamic and malonic acid [34]. Pg-soph-5-glu derivatives with molecular weight between 900 and 950 Da accounted for approximately 22%, derivatives with molecular weights between 950 and 1000 Da accounted for approximately 39%, and derivatives with molecular weights ≥1000 accounted for approximately 39% of the total anthocyanins present in the extract used. This distribution is consistent with the literature [34,35]. 

The passage of compounds through the skin occurs through diffusion; therefore, target molecules for transdermal delivery generally have a molecular weight of ≤500 Da (45). This is due to the assumption that molecular weight is a direct reflection of molecular size. The anthocyanins of elderberry are primarily simple glycosylated derivatives of cyanidin, which bears 2 hydroxyl groups on the B-ring; the molecular weight of these polyphenols range approximately 450–580 Da. The anthocyanins of red radish are predominantly larger, more complex derivatives of pelargonidin; they bear glycosylation on 2 sites of the chromophore as well as acylation with malonic acid and hydroxycinnamic acid derivatives (ferulic, coumaric, and sinapic: fer, cou, sin). Based on these assumptions, red radish anthocyanins were hypothesized to be unable to overcome the SC barrier. 

### 3.2. Detection and Quantification of Anthocyanins in Dermal Tissues

**Figure 1:**
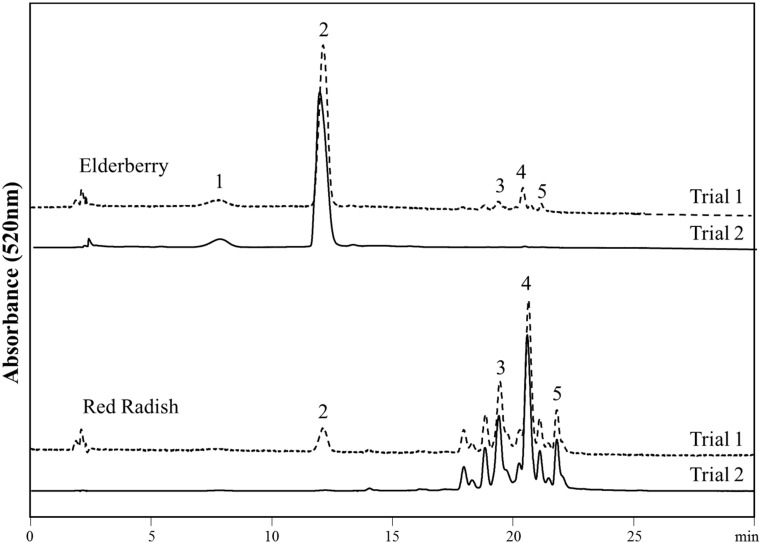
High-performance liquid chromatography (HPLC) chromatograms at 520 nm obtained from skin samples treated with ACN-lipstick formulations. Trial 1 chromatograms correspond to samples collected using an alternating pattern of lipstick formulations on the skin. Trial 2 chromatograms correspond to samples collected using a non-alternating pattern. Numbers correspond to peak identification shown in Table 1.

Anthocyanins were detected and quantified in samples prepared from both the ex vivo and in vivo tape strips by both ATR-FTIR and HPLC analyses. In general, the quantification of anthocyanins by HPLC analysis was in agreement with the results obtained from the ATR-FTIR analyses of the skin samples obtained from the volunteers (*R*^2^ = 0.99). Samples from the human Trial 1, in which the two lipstick formulations were applied to four test sites in an alternating pattern on the forearms, showed the appearance of elderberry anthocyanin peaks in the chromatograms from skin samples treated with red radish and the opposite. This demonstrated both lateral and transversal diffusion of anthocyanins through the stratum corneum. Representative chromatograms can be seen in Figure 1; numbers correspond to peak identification by HPLC-MS-PDA shown in Table 1. 

Peaks 1 and 2 are characteristic peaks for elderberry and peaks 3–5 are characteristic peaks for red radish anthocyanins. Interestingly, peaks 2–5 were observed in samples collected from both the elderberry and red radish skin treatment sites in Trial 1. In samples collected from lipstick treatment sites in Trial 2, in which the two lipstick formulations were applied to two test sites alternating with blank test sites on the forearms, only anthocyanins corresponding to the lipstick formulation applied were detected. However, peaks 2–5 (from both formulations) were identified in samples collected from untreated skin between treated test sites (not shown). This validated transversal diffusion occurred in Trial 1, rather than potential sample contamination. These peaks were below the LOQ; however, their identities were confirmed by mass spectrometry. These results demonstrated both penetration of anthocyanins through the skin and also their lateral diffusion of within the skin.

### 3.3. Characterization of ATR-FTIR Spectral Analysis

**Figure 2:**
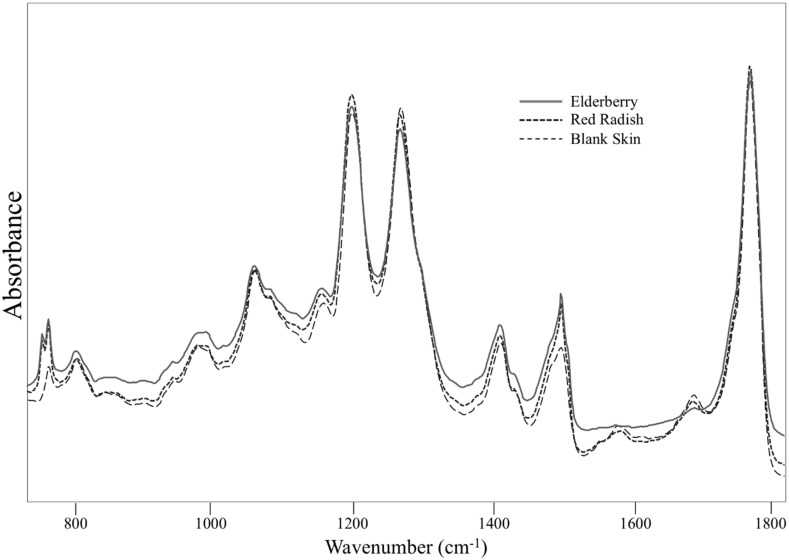
Representative attenuated total reflectance (ATR)-Fourier transform infrared spectroscopy (FTIR) spectra of samples from 700 to 1800 cm^−1^ after normalization to the most intense peak for each spectrum.

Representative FTIR spectra for the elderberry and red radish lipsticks on the skin, as well as spectra of blank human skin, can be seen in Figure 2. The spectral region from 700 to 1800 cm^−1^ was selected for comparison of the samples. The bands around 1743 and 1750 cm^−1^ were attributed to the characteristic carbonyl (C=O) stretch observed with skin and lipstick, respectively [36,37]. For IR characterization of the anthocyanins on the tape strips, the spectral region of 900–1160 cm^−1^ was assigned to the C-O stretching vibration of the sugar moiety of the glycosides present and to a lesser extent to the aromatic C-O stretching [38]. The band around 1445 cm^−1^ was attributed to the aromatic ring vibrations [38,39]. The region of 1150 and 1400 cm^−1^ showed multiple bands assigned to the C-O stretch and C-O-H bending of phenols, esters, carboxylic acid, and alcohols [39]. 

Attenuated total reflectance-Fourier transform infrared spectroscopy (ATR-FTIR) coupled with partial least squares regression (PLS-R) analysis was utilized for analysis of the samples due to the high sensitivity of the technique to subtle chemical variations and minimal sample preparation required [38]. Quality of the prediction was greatly improved by selecting specific wavenumbers (700–1700 cm^−1^) rather than using the entire spectra. The optimum number of factors giving the lowest standard error of cross-validation (SECV) values for the elderberry and red radish lipsticks were 6 and 5, respectively. Lower SECV values and higher correlation coefficients of validation (rVAL) values indicate better prediction ability and higher accuracy on the prediction of the desired variable [40]. The SECV values obtained with the model for the elderberry and red radish lipstick were 0.028 and 0.021, respectively. The rVAL were both 0.98, and thus the calibration models could be used to accurately predict the quantity of anthocyanins in the porcine and human skin samples. 

### 3.4. Determination of Anthocyanin Release and Penetration Kinetic Parameters

The initial release coefficient (K_r_) and the non-initial release coefficient (K_H_) of anthocyanins from the formations were both higher for elderberry than red radish (Table 2). These kinetic release parameters were calculated based on Fick’s first law of diffusion and Higuchi’s kinetic model, based on the cumulative amount of anthocyanin obtained from tape stripping and the concentration within the formulations. Due to the relatively short steady state period within the lipsticks, kinetic release parameters became limited to describing initial release rates of the anthocyanins from the formulas [14]. The further release kinetics are usually determined by the diffusion rate (D_V_) within the vehicle [41]. Zillich et al. (2013) reported higher K_r_ values for more hydrophilic polyphenols, such as catechins (K_r_ = 5.18 ± 0.12), and lower K_r_ values for more hydrophobic compounds, such as rutin (K_r_ = 1.65 ± 0.07) [14]. Release rates were proposed to be increased with decreasing molecular weight and increased hydrophilicity [14]. These two factors have been described as the most important considerations for release and skin permeation of ingredients from formulations [42]. Elderberry anthocyanins have greater hydrophilicity and smaller molecular weights when compared to red radish anthocyanins, possibly explaining the greater diffusion constants across the formulation. The lipophilic nature of the lipstick base may also improve the affinity of the anthocyanins to the SC as compared to the vehicle. Decreases in release rates of catechin, quercetin and rutin from topical cosmetic formulations have been reported when hydrophilic ingredients were included [43]. This suggests an increase in solubility of the flavonoids within the vehicle, and as a consequence, a decrease in the driving force of the permeation process occurred.

**Figure 3:**
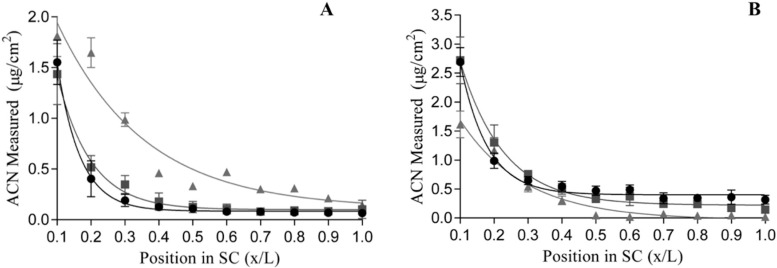
Relative position (depth) within the stratum corneum (SC, x/L) of mean anthocyanin amounts (μg/cm^−2^) ((**A**): elderberry; (**B**): red radish) as determined by HPLC in vivo (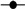), and PLS-R of FTIR spectra in vivo (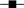) and ex vivo (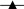). Means are shown as the average of triplicate samples from six participants. The lines are the best fit for Equation (A1) used to determine permeability and diffusivity of the formulas.

The relative position of the anthocyanins in the skin (x) was determined using Fick’s second law (Figure 3), based on the amount of anthocyanin per tape strip quantified as well as the area and thickness of the stratum corneum (L). Based on the fitting of Fick’s second law to experimental data obtained from the combined FTIR tape stripping technique, both the stratum corneum-vehicle partition coefficient (K) and the diffusivity of the anthocyanins as a function of the stratum corneum depth (D/L^2^) could be determined (Table 3). 

Estimation of the skin permeability coefficients of the anthocyanins within the stratum corneum can be seen in Table 4. The skin permeation coefficients (K_P_) for both elderberry and red radish were approximately two orders of magnitude smaller than their respective K_r_ values. The differences in these two coefficients highlight the difficulty of overcoming the SC barrier [44]. The partition coefficient between that of the formulation and the SC (K) showed a lower affinity of the anthocyanins for the skin than for the vehicle. When compared to the steady state flux (Jss), K indicates that the SC barrier must be overcome as a prerequisite for permeation of anthocyanins across the skin. Diffusion through the skin once past the rate-limiting SC should hypothetically arise much quicker and be independent of the vehicle composition [45]. The work by Bojanowski et al. (2013) supports this hypothesis, who reported the hypodermal delivery of anthocyanins from grape seed extract through a transbuccal membrane when incorporated into soy phospholipids [46]. Grape skin extract was able to permeate into both the dermis and epidermis when applied to the hypodermal side of the skin. Hypodermal delivery is one strategy for getting around the barrier function of the SC, and their work further suggests a beneficial role of anthocyanins for skin care.

The diffusivity (D/L^2^) of the red radish lipstick (Table 3) seems to suggest that the molecular weight of these compounds was not a deterrent to their diffusion in the skin. Several studies [47,48,49] have proposed folding of the anthocyanin molecule leading to stacking of the planar ring of the aromatic acid and the aromatic nucleus of the anthocyanin and π–π hydrophobic interactions. This folding (or intramolecular copigmentation) has also been proposed to account for the increased stability of acylated anthocyanins. More specifically, Giusti et al. (1998) [50] reported NMR results for spatial conformations of red radish anthocyanin molecules in which a close proximity was shown between the hydrogens of the acylating groups and the C4 of the aglycone structure, suggesting a sandwich-type folding of the acyl group over the pyrylium ring. Thus, once folded, the sizes of these molecules are more compact. This could contribute to the permeation of the anthocyanins within the SC, despite the large molecular weights of red radish anthocyanins. 

Another possible mechanism for the diffusivity of the red radish anthocyanins is the slightly amphiphilic nature of anthocyanins containing aromatic or aliphatic acylating groups [2,51]. Percutaneous absorption of compounds is lower for compounds that are extremely hydrophilic or hydrophobic, suggesting that a slightly amphiphilic nature may be an advantage for diffusion through the inhomogeneous layers of the skin below the SC [52]. Compounds that may not readily diffuse through the SC may be more inclined to spread radially within the bilayers [53]. The slightly amphiphilic nature of anthocyanins may also lend to lateral diffusion of the compounds, especially the acylated red radish anthocyanins. 

Anthocyanins from both lipstick formulations were found to penetrate the skin and were identified at depths relevant for overcoming the SC. In order for their beneficial properties to be observed, the anthocyanins must also reach the target skin site, such as the epidermis and dermis, and not permeate into the microcirculation. Catechins, EGCG, and quercetin have all been found to localize in the stratum corneum, viable epidermis and dermis [14,15,18,54], and these findings have been correlated with their health-promoting properties observed on the skin. Based on the evidence in the literature of similar health benefits of anthocyanins, it seems likely that they will also be preferentially located in these layers [6,20,55,56,57]. However, studies investigating their quantification in the epidermis and dermis, as well as the IC_50_ values necessary to exhibit these benefits in vivo, need to be performed.

## 4. Conclusions

Anthocyanins from elderberry and red radish incorporated into lipstick formulations were shown to permeate through the stratum corneum, which is the rate-determining phase for skin-penetrating compounds. The combined use of ATR-FTIR and PLS-R analysis was successfully able to quantify the anthocyanins removed by tape stripping of porcine and human skin with minimal sample preparation. These results were validated using HPLC-MS-PDA analysis, and a strong correlation between the two methods was observed. This allowed for the determination of release and skin permeation profiles for the anthocyanins from lipstick formulas. The use of Higuchi’s square root model was used to describe the diffusion of the anthocyanins within the lipsticks. The smaller hydrophilic compounds of elderberry were released and permeated within the skin at a faster rate; but importantly, anthocyanins from both elderberry and red radish were able to penetrate into the skin and reach depths relevant for their use as beneficial ingredients in skin care products. This work provides insight on the permeation of anthocyanin-type flavonoid compounds through the skin applied topically in a lipophilic delivery system and may contribute to the understanding of anthocyanin delivery in other similar topical applications.

## Figures and Tables

**Table 1:** Identities of anthocyanins from elderberry and red radish identified in skin samples after topical application by HPLC-PDA-MS. Numbers correspond to peaks found in Figure 1.

Peak	t_R_ (min)	Source	[M+]	Anthocyanin
1	8.3	Elderberry	581, 287	Cy-3-samb
2	14.4	Elderberry	449, 287	Cy-3-glu
3	23.6	Red radish	933, 271	Pg-3-fer-soph-5-glu
4	24.5	Red radish	989, 271	Pg-3-cou-soph-5-mal-glu
5	26.5	Red radish	1019, 271	Pg-3-fer-soph-5-mal-glu

**Table 2:** Kinetic parameters for the release of elderberry and red radish anthocyanins from lipstick formulations. All values are the mean ± standard deviation (*n* = 9). Statistical significance denoted by (*) (*p* ≤ 0.05), determined by two-way ANOVA.

Release from Lipstick Formulations			
**Formula**	K_r_^a^ × 10^2^ (cm h^−1^)	K_H_^b^ × 10^2^ (cm h^−0.5^)	D_V_^c^ × 10^3^ (cm^2^ h^−1^)
Elderberry	5.80 ± 0.11 *	7.33 ± 0.03 *	4.22 ± 0.02 *
Red Radish	4.04 ± 0.13 *	5.12 ± 0.06 *	2.06 ± 0.04 *

^a^ K_r_, initial release coefficient (Equation (A4)). ^b^ K_H_, Higuchi’s coefficient (Equation (A5)). ^c^ D_v_, diffusion coefficient within lipstick (Equation (A6)).

**Table 3:** Diffusivity and partition parameters obtained after application of elderberry and red radish lipsticks to the SC either in vivo human volunteers or ex vivo on porcine ear skin. All values are the mean ± standard deviation (*n* = 9). Statistical significance denoted by (*) (*p* ≤ 0.05), determined by two-way ANOVA.

Formula	Test System	K ^a^	D/L^2 b^ (h^−1^)
Elderberry	Ex vivo	0.229 ± 0.02	0.142 ± 0.05
	In vivo HPLC	0.224 ± 0.01	0.142 ± 0.04
	In vivo FTIR	0.224 ±0.02	0.144 ± 0.06
Red Radish	Ex vivo	0.204 ± 0.01	0.136 ± 0.07
	In vivo HPLC	0.209 ± 0.03	0.146 ± 0.02
	In vivo FTIR	0.207 ± 0.03	0.138 ± 0.01

^a^ K, Vehicle/SC partition constant; ^b^ D/L^2^, diffusivity coefficient within the skin (Equation (A1)), Figure 3.

**Table 4:** Estimated permeability coefficients (Kp) and steady state fluxes (Jss) of anthocyanins across the SC following lipstick application either in vivo in human volunteers or ex vivo on porcine ear skin (mean ± standard deviation, *n* = 9). Statistical significance denoted by (*) (*p* ≤ 0.05), determined by two-way ANOVA.

Formula	Test System	K_p_ × 10^4 a^ (cm h^−1^)	J_ss_^b^ (μg cm^2^ h^−1^)
Elderberry	Ex vivo	2.39 ± 0.59*	3.34 ± 0.11*
	In vivo HPLC	2.33 ± 0.72	3.26 ± 0.04
	In vivo FTIR	2.36 ± 0.37	3.30 ± 0.09
Red Radish	Ex vivo	2.02 ± 0.41*	2.93 ± 0.04*
	In vivo HPLC	2.00 ± 0.68	3.04 ± 0.05
	In vivo FTIR	2.09 ± 0.34	2.88 ± 0.15

^a^ Kp, permeation coefficient within the skin (Equation (A2)); t_L_, lag time for diffusion across the SC; ^b^ Jss, predicted steady state flux within the skin (Equation (A3)).

## Abbreviations

ACN	Anthocyanin(s)
ATR-FTIR	Attenuated total reflectance-Fourier transform infrared
HPLC-MS	High-performance liquid chromatography-mass spectrometry
Kp	Permeability coefficient
MW	Molecular weight
PLS	Partial least squares
PDA	Photodiode array
SC	Stratum corneum

## Appendix A

### Determination of Anthocyanin Penetration Profiles

After determining the thickness of the stratum corneum removed by each tape strip and quantifying the amount of anthocyanin in each tape strip normalized to the area, the diffusivity and solubility of the anthocyanins within the stratum corneum can be determined [13]. Fick’s second law of diffusion was used to predict concentrations of anthocyanin (*c_x_*) as a function of position (x) within the stratum corneum by applying the following Equation [31]:

(A1) $$c_{x}=c_{x=0}\left\{\left(1-\frac{x}{L}\right)-\frac{2}{\pi }\overset{\infty }{\underset{n=1}{\sum }}\frac{1}{n}\sin\left(\frac{n\pi x}{L}\right)\exp\left(\frac{-Dn^{2}\pi^{2}t}{L^{2}}\right)\right\}$$

The concentration of anthocyanin in the outermost layer of the stratum corneum (*c*_*x* = 0_) is divided by its concentration in the applied lipstick (*c_v_*), which provides the partition coefficient of the anthocyanin between the lipstick and stratum corneum (K), and the diffusion parameter (D/L^2^). D/L^2^ is the first-order rate constant of the anthocyanin diffusivity (D) in the stratum corneum to the thickness (*L*) squared of the barrier [28]. To apply Equation (A1) to determine these constants, some assumptions are made about the system [28]. The assumptions are: an infinite dose is applied, that the stratum corneum is a homogenous barrier and contains no permeant at *t* = 0, and that the diffusivity of the drug within the stratum corneum is slow compared to uptake by the cutaneous microcirculation, or a “sink” condition occurs for the permeant at the interface between the stratum corneum and the viable epidermis [58]. This allows for the SC to be considered the rate-limiting step in dermal permeability.

The values for D/L^2^ and *K*, combined with the corresponding SC thickness (*L*), make it possible to calculate the permeability coefficient (*K_P_*) from each of the anthocyanin-lipstick formulas tested using Equation (A2) [27]:

(A2) $$K_{p}=K\frac{D}{L^{2}}L=\frac{KD}{L}$$

In addition, knowing the *K_P_* and the concentration of the anthocyanins in the vehicle (*C_v_*), the steady state flux (*J_ss_*) across the SC can be estimated by:

(A3) $$J_{ss}=K_{p}C_{v}=\frac{KD}{L}C_{v}$$

The time necessary to reach steady state across the SC is related to the D/L^2^ by the classic diffusional lag-time (T_lag_ = L^2^/6D) [59]. Time to reach the steady state is generally regarded as about 2.7T_lag_ [59]. These Equations were applied for the determination of kinetic parameters of the anthocyanin-lipstick formulations in porcine ear SC.

Fick’s first law can be used to determine initial release rates of the anthocyanins from the formulations:

(A4) $$K_{r}=\frac{J_{ss}}{C_{v}}$$

where *K_r_* is the initial release coefficient, *J_SS_* is the steady state flux of the anthocyanin across the formulation and *Cv* is the initial concentration of anthocyanin in the formulation (μg cm^−3^).

Likewise, Higuchi’s kinetic model was used to understand the influence of the anthocyanin non-initial release kinetics from the lipstick formulas on the permeation kinetics within the SC:

(A5) $$\frac{Q}{C_{0}}=2\sqrt{\frac{D_{v}t}{\pi }}$$

where *Q* is the cumulative amount of permeated anthocyanin through a unit of membrane surface, *C*_0_ is the initial anthocyanin concentration within the vehicle, and *D_V_* is the diffusion coefficient within the vehicle over a given time t [14]. *D_V_* values can be derived from the plot of *Q*/*C*_0_ against the square root of time by the slope (*K_h_*) of the linear regression Equation:

(A6) $$K_{h}=2\sqrt{\frac{D_{v}}{\pi }}$$

Higuchi’s kinetic model determines the cumulative release profiles of the anthocyanins from the lipstick formulation [60]. *K_h_* describes the non-initial release rate and can be used to predict the time dependency of the release profile.
